# Association of obstetrical Hysterectomy with rising rates of cesarean section among women with Placenta Previa

**DOI:** 10.12669/pjms.39.1.6656

**Published:** 2023

**Authors:** Saima Ashraf, Saima Yasmeen Qadir, Arooj Fatima Khosa

**Affiliations:** 1 Saima Ashraf, FCPS (Obstetrics & Gynecology), Department of Obstetrics & Gynecology, Nishtar Medical University, Multan, Pakistan; 2 Saima Yasmeen Qadir, FCPS (Obstetrics & Gynecology), Department of Obstetrics & Gynecology, Nishtar Medical University, Multan, Pakistan; 3 Arooj Fatima Khosa, FCPS (Obstetrics & Gynecology), Department of Obstetrics & Gynecology, D.G. Khan Medical College, Dera Ghazi Khan Pakistan

**Keywords:** Cesarean section, hysterectomy, placenta previa

## Abstract

**Objective:**

To analyze the impact of previous cesarean section on rates of obstetrical hysterectomy among women with placenta previa.

**Methods:**

A retrospective cohort study done at the Department of Obstetrics & Gynecology, Nishtar University Hospital, Multan from January 2017 to December 2021. A total of 374 women aged between 20 to 50 years with diagnosis of placenta previa who underwent cesarean delivery were included. Age, BMI, area of residence, gestational age, gravidity status and past history of cesarean section were noted from medical records of all women. Relationship of previous number of cesarean sections and with obstetrical hysterectomy was noted among women with placenta previa.

**Results:**

In a total of 374 women, mean age was 33.45±7.3 years. Mean gestational age was 37.4±3 weeks. Obstetrical hysterectomy was done in 110 (29.4%) women. Significant association of gravidity status was noted with hysterectomy as 95 (86.4%) women with hysterectomy had gravidity status ≥3 in comparison to 197 (74.6%) with no hysterectomy (p=0.030). Number of cesarean section was found to have significant association with obstetrical hysterectomy as 55 (50.0%) women with obstetrical hysterectomy reported ≥3 previous cesarean section versus 29 (11.0%) with no obstetrical hysterectomy (p<0.001).

**Conclusion:**

Strong association of previous number of cesarean section and obstetrical hysterectomy was found with placenta previa. There should be protocols for early identification and prediction of adverse outcomes among women with placenta previa with past history of cesarean section.

## INTRODUCTION

Placenta previa is described as a condition in pregnancy where placenta implantation occurs abnormally in the lower uterine segment that could cover the internal cervical so partially or completely.^1^ The global prevalence of placenta previa is calculated to be around 4/1,000 live births.^2^,^3^ Recent local data has revealed prevalence of placenta previa around 1.9%.^4^

Massive hemorrhage, obstetrical hysterectomy and maternal death are some of the major and known complications of placenta previa.^5^,^6^ Researchers in the past have shown that women presenting with placenta previa and past history of cesarean section (CS) are at increased risk of placenta accreta. Recent data has shown that risk of placenta previa-accreta has been rising in the past few decades which could be credited to rising rates of CS globally.^7^ The rise in CS rates is more evident among urban populations and women above 35 years of age.^8^,^9^

The chances of maternal and perinatal morbidity are increased among women with placenta previa but not much research has been done to find out relationship of hysterectomy with past CS history in women with placenta previa. Our aim was to analyze the impact of number of previous cesarean section on rates of obstetrical hysterectomy among women with placenta previa. The findings of this study were thought to help clinicians predict the risk of obstetrical hysterectomy among women having placenta previa who present with previous history of CS which may assist us in devising some strategies to reduce cesarean section rates among these women.

## METHODS

This retrospective cohort study was conducted at the “Department of Obstetrics & Gynecology, Nishtar University Hospital, Multan” from January 2017 to December 2021. Approval from “Institutional Ethical Committee” was acquired (letter no: 71, dated: 16-12-2017). A total of 374 women aged between 20 to 50 years with diagnosis of placenta previa who underwent cesarean delivery were included.

Age, BMI, area of residence, gravidity status, gestational age and past history of cesarean section were noted from medical records of all women. Obstetrical hysterectomy was considered as the main outcome. Relationship of previous history of cesarean section with obstetrical hysterectomy was noted among women with placenta previa.

SPSS version 26.0 was employed for data handling and analysis. Frequencies and percentages were used to represent categorical variables. For numeric data, mean and standard deviation (SD) were calculated. Comparison of qualitative data was made using chi-square test while quantitative data was compared utilizing independent sample t-test. P value ≤ 0.05 was taken as significant.

## RESULTS

In a total of 374 women, mean age was 33.45±7.3 years while 165 (44.1%) women were aged between 31 to 40 years. There were 214 (57.2%) women from rural areas of residence. Mean BMI was 25.7±2.8 kg/m^2^ (ranging between 22-32 kg/m^2^) while 186 (49.7%) women had BMI below 25 kg/m^2^. There were 292 (78.1%) women who had gravidity status ≥ 3 (ranging between 1 to 9). Previous history of lower segment CS was noted in 188 (50.3%) women. Table-I is showing characteristics of all women.

**Table-I:** Baseline Characteristics of Women (n=374)

Characteristics		Number
Age Groups (Years)	20-30	110 (29.4%)
	31-40	165 (44.1%)
	41-50	99 (26.5%)
Area of Residence	Rural	214 (57.2%)
	Urban	160 (42.8%)
BMI	<25	186 (49.7%)
	25-29.9	141 (37.7%)
	≥30	47 (12.6%)
Gestational Age (weeks)	<37	115 (30.7%)
	≥37	259 (69.3%)
Gravidity Status	1	37 (9.9%)
	2	45 (12.0%)
	3	292 (78.1%)
Number of Cesarean Section in the Past	None	186 (49.7%)
	1	53 (14.2%)
	2	51 (13.6%)
	≥3	84 (22.5%)

Obstetrical hysterectomy was done in 110 (29.4%) women. No significant association of hysterectomy was noted with different age groups (p=0.453), area of residence (p=0.483) or BMI (p=0.122). Significant association of gravidity status was noted with hysterectomy as 95 (86.4%) women with hysterectomy had gravidity status ≥3 in comparison to 197 (74.6%) with no hysterectomy (p=0.030). Comparison of characteristics of women with respect to obstetrical hysterectomy is shown in Table-II.

**Table-II:** Comparison of characteristics of women with respect to Obstetrical Hysterectomy.

Characteristics		Obstetrical Hysterectomy		P-Value

		Yes (n=110)	No (n=264)	
Age Groups	20-30	29 (26.4%)	81 (30.7%)	0.453
	31-40	54 (49.1%)	111 (42.1%)	
	41-50	27 (24.5%)	72 (27.3%)	
Area of Residence	Rural	66 (60.0%)	148 (56.1%)	0.483
	Urban	44 (40.0%)	116 (43.9%)	
Gestational Age (weeks)	<37 (115)	35 (31.8%)	80 (30.3%)	0.773
	≥37 (259)	75 (68.2%)	184 (69.7%)	
BMI (kg/m^2^)	<25	60 (54.5%)	126 (47.7%)	0.122
	25-29.9	33 (30.0%)	108 (40.9%)	
	≥30	17 (15.5%)	30 (11.4%)	
Gravidity Status	1	5 (4.5%)	32 (12.1%)	0.030
	2	10 (9.1%)	35 (13.3%)	
	≥3	95 (86.4%)	197 (74.6%)	

Previous history of CS was found to have signification association with hysterectomy as 55 (50.0%) women with hysterectomy reported ≥3 previous cesarean section in comparison to 29 (11.0%) patients without hysterectomy (p<0.001). In comparison to no past history of CS, there was 1.9 fold increase in the risk of hysterectomy with one cesarean section, 2.6 fold with two cesarean sections and 5.5 fold increase with three or more cesarean sections. Association of number of previous CS with obstetrical hysterectomy is shown in Table-III.

**Table-3:** Association of number of previous cesarean section with Obstetrical Hysterectomy (n=374).

Number of Previous Cesarean Section	Obstetrical Hysterectomy		P-Value

	Yes (n=110)	No (n=264)	
None	10 (9.1%)	176 (66.7%)	<0.001
1	19 (17.3%)	34 (12.9%)	
2	26 (23.6%)	25 (9.5%)	
≥3	55 (50.0%)	29 (11.0%)	

## DISCUSSION

Mean age of the women with placenta previa was 33.45±7.3 years while 44.1% women were aged between 31 to 40 years and 26.5% between 41 to 50 years. Local data evaluating cases of placenta previa undergoing cesarean delivery noted mean age of women to be 28.6±4.5 years which is somewhat lower than what was noted in this study.^10^ Sindiani et al. from Jordan analyzing cases of placenta previa revealed mean age of the women to be 32.1±4.9 years which is close to what we noted in this study.^11^

We noted that 50.3% women had previous history of cesarean section. Wasim T et al.^4^ from Lahore analyzing women with placenta previa showed that past history of CS was noted in 84.2% which is much higher than what was found by us. Another local study showed that history of cesarean section was noted in 54.1% women with placenta previa which is quite close to what was noted in this study.^10^

In the present study, cesarean hysterectomy was done in 29.4% women with placenta previa. Maqsud M et al. from Lahore reported rates of hysterectomy to be 19.2% among women with placenta previa.^10^ Liu B et al. from China found hysterectomy rates of 11.4% among accompanying placenta with past history of cesarean section.^12^ Some models like “hysterectomy index in placenta previa with prior cesarean (HIP)” are also in practice to predict adverse maternal outcomes which can help in prediction of pre-surgical risk of cesarean hysterectomy among women having placenta previa.^12^

In this study, previous history of CS had signification association with hysterectomy as 47.3% women with hysterectomy reported ≥3 previous cesarean section in comparison to 11.0% of patients with no hysterectomy (p<0.001). Likewise, 9.1% women with hysterectomy performed reported no history of previous cesarean section in comparison to 66.7% cases where hysterectomy was not performed (p<0.001). The risk of placenta previa has been reported to increase by 0.28–2% in patients who have undergone at least one CS in a meta-analysis including 36 trials.^13^,^14^ Another cohort study spanning 9-year follow up involving primiparous women revealed incidence of placenta previa as 5.3/1000 births.^15^ However, there is also data that do not support this finding, especially for placenta previa.^16^ In a recent study from Romania, Gica N et al. found that placenta related abnormalities were the leading cause of emergency peripartum hysterectomy and found strong relationship of increased rates of past history of CS with emergency peripartum hysterectomy.^17^ Identification of placenta previa prior to cesarean section is challenging as many of these cases are not diagnosed prior to cesarean section. Absence of pre-operative diagnosis of placenta previa can lead to higher rates of maternal morbidity and mortality.^18^

### Limitations of the Study:

Retrospective study had its own limitations. As this was a single center study, our findings cannot be generalized. We were unable to find out other most commonly occurring maternal adverse outcomes due to lack of availability of data. We also could not evaluate fetal outcomes and its relation with past history of cesarean sections performed among women with placenta previa.

## CONCLUSION

In our study, strong association of previous number of cesarean section and obstetrical hysterectomy was found among women with placenta previa. Due to associated maternal morbidity and maternal mortality, there should be protocols for early identification and prediction of adverse outcomes among women with placenta previa with past history of cesarean section.
